# Analysis of the Selection Impact of 2D Detectors on the Accuracy of Image-Based TLS Data Registration of Objects of Cultural Heritage and Interiors of Public Utilities

**DOI:** 10.3390/s20113277

**Published:** 2020-06-09

**Authors:** Jakub Markiewicz, Dorota Zawieska

**Affiliations:** Division of Photogrammetry, Remote Sensing and Spatial Information Systems, Faculty of Geodesy and Cartography, Warsaw University of Technology, Pl. Politechniki 1, 00-661 Warsaw, Poland; Dorota.Zawieska@pw.edu.pl

**Keywords:** image-based TLS registration, detectors, quality assessment, ASFT, SURF, FAST, BRISK, CenSurE, cartographic projections

## Abstract

The aim of this article is to present the influence of detector selection for the image-based Terrestrial Laser Scanning (TLS) registration method. The presented results are the extended continuation of investigations presented in the article, ‘The Influence of the Cartographic Transformation of TLS Data on the Quality of the Automatic Registration’. In order to obtain the correct results of the TLS registration process, it is necessary to detect and match the correct tie points, which are evenly distributed across the entire area. Commonly, for TLS data registration manually or semi-manually corresponding points are detected. However, when large, complicated cultural heritage objects are investigated, it is sometimes impossible to place marked control points. The only possibility of resolving this problem is the use of image-based TLS data registration. One of the most important factors that influences the quality and ability to use it correctly, is accurate selection. For this purpose, the authors decided to test three blob detectors ASIFT, SURF, CenSurE, and two point detectors FAST and BRISK. The results indicated that selection depends on two factors: if the time required for data processing is not important, the ASIFT algorithm should be used, which allows for full registration, but if not, a combination of other algorithms with results supervision should be considered.

## 1. Introduction

Terrestrial Laser Scanning (TLS) has been widely applied in the inventory of different architectural or industrial objects, both for the creation of conventional architectural documentation (such as vector drawings or cross-sections), as well as for high-resolution orthoimages or 3D models [1,2,3,4,5,6].

When complicated and large objects are investigated and processed, the acquisition of all the elements in one dataset is practically impossible, due to the presence of so-called blind spots, complexities of the object and different measurement errors, such as mixed-edge effect, intensity noises and the influence of the indicate angles [6,7,8,9,10,11,12]. The TLS data are acquired in the local reference system of an instrument; therefore, in the case of a large number of scans (which are required for large, complex objects), it is necessary to register scans in one defined reference system. Hence, it is necessary to prepare the plan of the TLS positions, because it influences the method and pipeline of the point clouds registration. This process relies upon point clouds registration in the defined reference system, such as the global coordinate system or the internal coordinate system, related to one of the scans (the so-called reference scan).

Several methods of TLS data registration exist, which may be generally divided into target-based and feature-based methods and are discussed in many publications [2,9,11,12,13,14,15]. In general, this method is based on the corresponding points between two or more datasets, but the main differences may be found in the process of locating and matching these corresponding points. In order to determine the relationship between the local instrument and the global reference system, Equation (1) is used [14,16,17]:

(1) $$M_{\mathrm{ext}}=R_{\omega \varphi \kappa }*M_{\mathrm{int}}+T$$

where $M_{\mathrm{ext}}$ is the vector of the point coordinates in the reference system, $M_{\mathrm{int}}$ is the vector of the scanner coordinates of points in the local system, T is the translation vector, $R_{\omega \varphi \kappa }$ is the rotation matrix: As a result of the data orientation process, exterior orientation elements are obtained for each scan, i.e., to determine the position of the scanner system center in the assumed reference system and angles of rotation, which are then used to transform the point cloud.

In order to determine the registration parameters, two main methods are applied: (1) point-based: these are target-based methods, based on matching point clouds on the basis of marked control points and ICP (Iterative Closest Point)/SLAM (Simultaneous Localization and Mapping) methods, based on matching groups of points to reference planes, point clouds, or shapes and [14,15,17,18,19] (2) feature-based: a process for matching point clouds which utilizes features detected in point clouds, such as curvature, edges, planes, etc., also known as the Structure-from-Motion (SfM) approach [18,20,21].

Recent research works relating to TLS data orientation focused on feature-based matching and are based on raster images, generated from scans. Corresponding geometric features, serving as a detection of elements of transformation are recognized on those images; detection of tie points is performed by means of Computer Vision (CV) algorithms and the Structure-from-Motion (SfM) method [13,22,23,24,25,26,27]. The key stage which influences the completeness and accuracy of TLS data automatic registration, based on the SfM approach, is the selection of appropriate 2D detector for the correct selection of well distributed tie points. This affects the quality of TLS data registration, as well as full automation of the registration process and a reduction in the time of finding and matching corresponding time [13,23,27].

When Cultural Heritage interiors are measured, it is often impossible to use the target-based registration method, due to the fact that, touching historical surfaces or placing any signals on them is prohibited, rendering the marking of control points difficult. In addition, the geometry of the interiors of cultural heritage objects is usually complicated; it is characterized by diversified depth and texture. Historical surfaces are often gilded, covered with decorative fabrics, and characterized by architectural detail. Since they are irreplaceable, it is recommended to use non-invasive TLS measuring and registration methods. The use of algorithms in the automated identification of the characteristic points of raster data, allows for an improvement in the methodology of data orientation and processing, in order to develop photogrammetric products.

The aim of this paper is to determine the influence of the selection of the 2D detector on the accuracy and completeness of TLS data registration. In the previous article [27], the authors proposed a novel method of TLS data registration, based on different cartographic transformation and two commonly used point (FAST) and blob (SURF) detectors. It should be stressed that choosing adequate detector effect the completeness of the data registration, time of computation and quality of registration. This article is a continuation of previous work and within it, the compilations of the effectiveness of different blob and point detectors are presented, not only of the commonly used SURF and FAST detectors but also of ASIFT (the detector which considers the influence of affinity); CenSurE (center–surround filters), used for real-time calculations and decreasing calculation time; and BRISK (the extended version of the FAST algorithm, based on image pyramids), for automatic TLS data registration with three different 2D point clouds representations (spherical images, orthoimages, and rasters in Mercator representation). For that purpose, the interiors of buildings with historical surfaces of a decorative structure and design were chosen; for such objects, it is not possible to distribute points utilized in the data orientation process using the target-based method. Additionally, for independent analysis, the interiors of public utilities (office and empty shop in shopping mall) were also chosen.

This paper was divided into five main sections; in Section 2, the state-of-the-art SfM approach was used and the key-point detectors were presented. Section 3 contains a description of the test sites and approach used, as well as the data analysis method. In Section 4, the results of the detector assessments were summarized. In the conclusion (Section 5), future works were proposed, and the possibilities and limitations of using different 2D detectors were summarized.

## 2. Related Works

The current research works concerning TLS data orientation are based on the method-based approach, the SfM method and on point clouds, converted into the raster form.

Conversion of a point cloud into a spherical image is the method mostly applied and is implemented in many commercial software tools [13,24,28,29,30]. For the generation of spherical images, raw data are used. This allows for the generation of raster with the highest resolution and without the interpolation of new values of pixel coordinates. Due to the fact that spherical images are encumbered with geometrical deformations, another representation of the point clouds is required. For that reason, the “virtual photograph”, (based on the collinearity equation) orthoimages or raster in cartographic transformation, such as the Mercator projection (which allows the projection of upper fragments of a scan with smaller geometric deformations), could be used.

### 2.1. Foundations of the Operations of Detectors and Descriptors

When the SfM [31] method is used, characteristic features (key points) are individually detected for each processed photograph/raster data and in the next steps, they are matched at each successive stage. They may cover points, edges, lines, or entire regions; thus, they create a group of the so-called-local features; several local features create the global structure. Determining tie points is a three-stage process: (1) initially, reference areas are detected using detectors, according to an assumed key, (2) then, their characteristic features are outlined by descriptors, and (3) finally, the matching of a point is carried out, based on the methods of statistical matching of features [13,20,23,31].

Invariant features are determined, which form the basis for comparing points in different rasters. The detection and description of features for each characteristic point is an important element of the process of detection of homologous points, because the final recognition of points as tie points is performed by means of matching their relative descriptors in the process of data orientation. For that purpose, two approaches are usually applied: the Approximate Nearest Neighbor-Based Point Matching [32] and Brute Force matching [33]. In the next subsection, blob (SURF, ASIFT, CenSurE) and point detectors (FAST and BRISK) used in this investigation are described.

#### 2.1.1. FAST Detector

The idea of operating the FAST detector (Features from Accelerated Segment Test) [34], presented by Rosten and Drummond in 2006, is based on the assumption that characteristic points have a clearly defined position and that they are the media of easily recognizable information, which allows for their explicit detection in neighboring images. The advantage of the FAST detector is the image processing speed, since it was designed in order to detect tie points in real time, for example in cases when the orientation of photographs is performed by means of a SLAM algorithm.

Operations of the FAST algorithm consist of five main steps: (1) selection of an appropriate pixel, which will be analyzed in relation to its membership in a group of points, considered as corners. The intensity value of the determined point is marked Ip; (2) determination of an acceptable threshold value, below which value points are rejected; (3) definition of a circle with a radius of 16 pixels, for which pixel values will be analyzed; (4) definition of an initially selected point as a corner—if n (defined number) pixels with a greater intensity or equal Ip are located within its neighborhood, (i.e., within the circle of the perimeter of 16 pixels), 12 of them are selected; (5) selection and comparison of pixels marked by the numbers 1, 3, 5, and 13, in order to accelerate the operations of the first stage of the algorithm. When the first two pixels are darker or brighter than the pixel being compared, the algorithm skips to the next step, i.e., to compare two successive pixels. In the case of a pixel which may be a possible corner, it is necessary to check whether the three surrounding pixels are brighter or darker. If both presented conditions are satisfied, successive pixels are compared, in order to check the accuracy of the assumed hypothesis.

#### 2.1.2. BRISK Detector

Another detector applied to identify characteristic points on photographs is the BRISK (Binary Robust Invariant Scalable Key points) detector [35]. Its operation is similar to the approach applied in the FAST detector, and the difference is in the method of searching for characteristic points. In the case of the FAST detector, points are searched for in the full resolution image, while in the case of the BRISK detector, in different image pyramids. The term “image pyramid” corresponds to the recording of an image, which has been processed using a Gauss filter, which eliminates every nth pixel, depending on the assumed level of details and the resolution of an analyzed image. Criteria which decide on the qualification of characteristic points, are the same as in the case of the FAST algorithm (Section 2.1.1).

The image pyramid applied in the BRISK algorithm consists of n c_i_ levels which include n sublevels d_i_, for i = {0, 1, ..., n − 1} for the value of n = 4. Certain levels result from re-sampling every second pixel of the previous level of the image pyramid, starting from the original image. The first sub-area, do, is created based on a generalization of the original image, co, by means of the coefficient 1.5. To detect characteristic points, a mask 9–16 is applied; it is used for processing 9 out of 16 neighboring pixels. The point detection coefficient is the same as in the case of the FAST algorithm (Section 2.1.1).

In order to distinguish characteristic areas, the FAST 9-16 detector is used separately at each level (area) and subarea, with the same boundary threshold I. The characteristic points detection process is performed in a similar way to the FAST detector, with the exception of corner detection, which is performed at different levels of the image pyramid.

#### 2.1.3. ASIFT Detector

The ASIFT (Affine Scale-Invariant Feature Transform) detector is a modified version of the commonly applied SIFT (Scale-Invariant Feature Transform) algorithm [36]. It is assumed that in SIFT operations, the points detection process is independent of the change of scale and rotation of photographs around the Z axis and that the translation [37] of the ASIFT algorithm is an extended version of the SIFT algorithm, which recognizes affinities, resulting from the differences in the scale of projection along the ox axis in relation to the oy axis.

The operations of the ASIFT algorithm may be described in the following stages: transformation of each image in which the influence of distortion resulting from the affinity is simulated and compared with the processed images which utilize the method of the SIFT algorithm [36].

When the algorithm for matching features detected by the SIFT descriptor is applied, errors caused by matching similar features of points, which ultimately are not tie points, may occur. The ASIFT algorithm which compares many pairs of processed images, excludes the use of a standard approach to matching features applied in the SIFT algorithm, due to an accumulation of gross errors. Therefore, the authors of the ASIFT algorithm have proposed a new, extended approach which assumes that the feature matching criterion should be compatible with the epipolar geometry. For that purpose, the ORSA solution is applied [38] which is recognized as the most accurate and resistant to gross errors and is at variance with the approach applied in the RANSAC algorithm. The ASIFT algorithm uses both features detected and described by the SIFT algorithm, taking account of the affinity. Such a redundancy of information allows an increase in processing accuracy.

#### 2.1.4. SURF Detector

The SURF detector (Speeded Up Robust Features) [39] was presented in 2006 by Bay et al. This detector is a modification of the SIFT detector. The authors assumed that the new algorithm would operate faster than the SIFT algorithm, and they based it on the use of the Hessian matrix. SURF operations consist of four stages, the first two of which concern the detection of characteristic points and the remaining two stages refer to the description of points using a descriptor. The basic assumptions of the SURF algorithm include: (1) calculation of an integral image; (2) location of key points; (3) assigning of key points; and (4) generation of descriptors.

The location of key points is carried out by means of the Hessian matrix, which significantly accelerates the search for tie points and improves the accuracy of their detection. A detected characteristic point corresponds to the maximum of a determinant of the matrix H (x, y, σ). A 9 × 9 filter size is used to determine this point; σ values for the lowest level of the pyramid are considered in that window. The processing of images using the SURF detector is connected to the use of rectangular filters and integral images; their basic advantage is the lack of necessity of an iterative application of the same filter in many photographs. As a result, it is possible to apply different filters at different levels of the image pyramid, and therefore, the (rectangular) filter size scaling process may substitute image scaling through elimination of particular pixels. At the first stage, the mask value of 9 × 9 and the scale value of 1.2 are assumed; those values correspond to the parameter of Gauss filtration σ = 1.2. Subsequently, the mask size is increased, to the size of 9 × 9, 15 × 15, 21 × 21, 27 × 27, etc. In the case of larger scales, the mask should be increased by adding value constantly until it has increased two-fold (n × 6); similar changes are applied to the value of the σ coefficient, for example, with regard to the filter 27 × 27, the value of σ will be increased 3 × 1.2 = 3.6.

In order to detect the characteristic points in an image and in the derivative images at other scale areas, 3 × 3 × 3 neighborhood are analyzed, and then the maxima of the Hessian matrix determinant are determined; they are interpolated on different scales, based on the Brown and Lowe method [40].

#### 2.1.5. CenSurE Detector

Another method of detecting characteristic points by means of blob descriptors is the use of the CenSurE [41] detector, proposed by Agrawal, Konolige, and Blasa in 2008. The basic assumption of operations performed by that detector is based on processing each pixel with the full image resolution. The authors proved that the maximum value of the Laplace operator is constant at many levels of image pyramids. On that basis, they proposed to apply that relationship within the entire area. They also presented a new class of filters (center–surround, or CenSurE, filters) that may be calculated independently on a scale and, therefore, may be used for real-time calculations. Operations of CenSurE are very similar to the operations of the SIFT and SURF algorithms.

The main feature which distinguishes the CenSurE algorithm is the use of the full image resolution for different filtration parameters. SIFT and SURF algorithms are based on the calculation of values of determinants at every scale (pyramid level) but they do not determine the maximum values independently for each of the scales. The CenSurE algorithm uses an approximation of the Laplace operator to determine the maximum values in the photograph, in the center–surround form; this allows for an acceleration of the process of calculations and an elimination of the influence of rotation (opposite to the DoB—Difference of Boxes—Hessian, which is utilized in the SURF algorithm). Besides, CenSurE is based on the Harris corner detection algorithm and produces better results than the hessian determinant.

#### 2.1.6. SIFT Descriptor

In order to match characteristic points in several photographs, it is necessary to describe their features based on their neighborhood [40]. This is performed by descriptors which enable the determination of invariant features, forming the basis for comparing points in different photographs.

In order to unify the descriptions of characteristic points, it was decided in this dissertation to utilize one descriptor for each detector. For that purpose, the operations of the SIFT descriptor were presented [40].

The operations of the SIFT descriptor consist of two key stages: (1) calculation of the gradient (scale) and orientation of each point within the neighborhood of a key point and (2) determination of a 128 element vector of features (a descriptor).

The orientation of key points is determined on the basis of one of the Gaussian images, the scale of which corresponds to the scale of a given key-point. For each image point, the gradient module and orientation are calculated.

All features of the key-point are measured in relation to the determined orientation; as a result, the description is independent of rotation. In the SIFT algorithm, the gradient module and orientation are considered within the neighborhood of 16 × 16 for a given key point. Then, this area is divided into regions of 4 × 4 size, in which the resultant histograms of orientation are re-created.

Based on the particular points of the modules, the resultant gradient module for eight orientations is determined within each area. Thus, the point feature descriptor is a vector consisting of 4 × 4 × 8 = 128 elements. The vector is normalized in order to reduce the influence of illumination.

The detection and description of features for each characteristic point is an important element of the process of tie points detection in digital images. The next stage of considering points as tie points in the process of image data orientation is their mutual matching. In this project the Approximate Nearest Neighbor-Based Point Matching [32] was used.

## 3. Materials and Methods

### 3.1. Overview of the Approach

The proposed methodology of analysis of blob and corner detectors is part of the multi-stage process of automatic TLS data registration; it is based on the original software and consists of (1) data conversion to the raster form; (2) aligning of pairs of raster TLS data for all possible combinations, based on FAST, BRISK, CenSurE, SURF, and ASIFT; (3) analysis of the quality of relative orientation of processed pairs; (4) selection of multi-criteria detector/detectors; and (5) final bundle adjustment process. In the previous article [27], the authors proposed the novel methodology of TLS data registration, which was based on the SfM approach (FAST and SURF detectors) and point clouds in three raster forms (spherical and Mercator projection and orthoimages). This article is a continuation of the previous investigation; however, three commonly used blob and point detectors (ASIFT, CenSurE, BRISK) in the same four test sites were tested.

In order to perform this experiment, the original application, based on different function libraries was applied, and to reliably evaluate the use of each algorithm, the authors also proposed a series of coefficients, which determined the accuracy and usefulness of each analyzed detector, including:

1. the time of detection of characteristics/key points;
2. the completeness of data registration;
3. the number of detected control and check points;
4. the orientation accuracy of control, natural, and marked check points;
5. the distribution of control and check points.

Then it was possible to determine which detector, with which method of 2D point cloud representation, allowed the highest accuracy to be obtained for TLS data.

In order to verify the aforementioned assumption, point clouds acquired by the Z + F 5003 and 5006 h laser scanner, characterized by a different measurement accuracy, were chosen. The data processing process consisted of the following stages:

1. conversion point from Z+F binary to ASCII file using the Z+F Software Development KIT (SDK);
2. generation of intensity raster in the spherical, orthoimages and Mercator projection with maps of XYZ coordinates in the original application, based on the ArcPy (Esri, Redlands, CA, USA, 2014) and Laspy (https://github.com/laspy/laspy, 2019) libraries;
3. detection of characteristic points using blob (SURF, CenSurE, ASIFT) and point detectors (FAST, BRISK) on point clouds in three different raster projections in the original software, based on the C++ and OpenCV library. In order to determine the number of combinations of possible pairs of TLS raster images, the method of permutation without repetition (2) was used.
  (2) $$l*\left(\begin{array}{c} n\\ k \end{array}\right)=\frac{l*n!}{k!\left(n-k\right)!}$$
  where k = 2 (a pairs of scans), n—the number of all scans and l—the number of planes used for orthoimages generation (the number of walls, the calling and the floor), in the case of the raster in spherical and Mercator projection l = 1;
4. description of all detected points by SIFT descriptor (OpenCV library);
5. computation of XYZ coordinates based on the XYZ maps (ArcPy library);
6. matching of possible tie points (on pairs of rasters) in relation to values obtained from the description process with the use of the Approximate Nearest Neighbor-Based Point Matching (OpenCV library) algorithm (Triangulation);
7. verification of the matching process in the iterative pre-bundle adjustment process with point filtration (RANSAC method; Armadillo library);
8. iteration of filtration point with three thresholds, 0.5 m, 0.1 m, and 0.01 m;
9. analysis of the number, deviations of points, and the distribution of detected tie points. If points are distributed across the whole area and the values of the deviations of points are ≤0.02 (for Test Sites I–III) and 0.04 m (for Test Sites IV), the detected and matched points are used in the final bundle adjustment process (Stage 7.3). Otherwise, those points are used to compute the approximate exterior orientation, which is treated as the first approximation of the Iterative Closest Point (ICP) method;
10. division of the tie point into the Octree form (Open3D library); analysis of the number of tie points in the node of each Octree; if the number is higher than six, divide the points into the control and check points;
11. determination of the approximate elements of the exterior orientation and final analysis of RMSE values on control and check points, used in Step 8;
12. final bundle adjustment for all pairs of scans (TLS) to one defined reference scan;
13. analysis and graphical presentation of results carried out using Matplotlib library.

### 3.2. Characteristics of Raw Data and Selected Test Sites

In order to verify which of the tested detectors is useful for the interior registration of automatic TLS point clouds, two decorated historical chambers at the Museum of King Jan III’s Palace at Wilanów, an office, and an empty shop at the shopping mall were selected. Choosing not only cultural heritage interiors, characterized by a diversified structure and surface geometry, enables an analysis of the effectiveness and accuracy of detecting and matching key points used as tie points. TLS data, used in these investigations, were acquired by two phase-shift scanners Z + F 5003 (Test Site I) and Z + F 5006 h (Test Sites II–IV) from different positions and heights. For the independent quality assessment, marked check points (that was not used for orientation parameters determination) on three of the four test sites were measured in the local coordinate system related to the reference scan (separately for each pair of scans). In order to determine the XYZ coordinates of the marked points, it was necessary to use the algorithm implemented in Z + F LaserControl software, which identifies the center of the target automatically based on the initial part of the point cloud target area.

#### 3.2.1. Test Site I “The Queen’s Bedroom”

**Figure 1:**
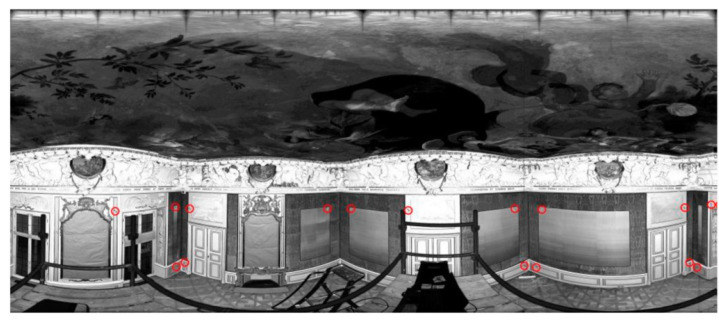
The point cloud in the Mercator projection of Test Site I: “The Queen’s Bedroom” with marked check points (red circles) [27].

Test Site I is a complex geometric chamber in which many ornaments, bas-reliefs, and facets exist. Additionally, there are mirrors in gold frames, a decorative fireplace, and fabrics, etc., hanging on the walls (Figure 1). In Figure 1, the marked check points (red circles) used for the independent quality assessment are shown.

**Figure 2:**
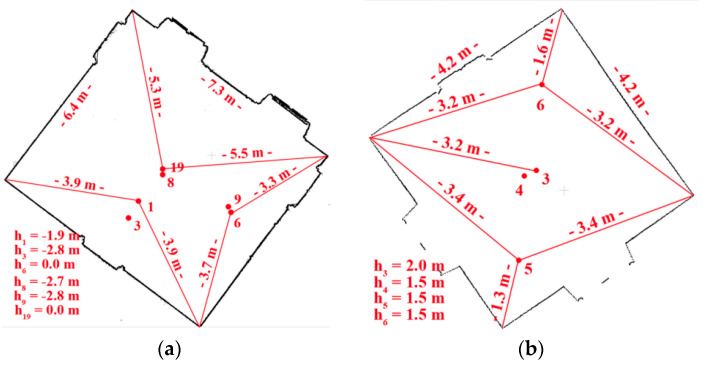
The floor plan with marked Terrestrial Laser Scanning (TLS) scanner positions with distances to the nearest walls for (**a**) Test Site I: “The Queen’s Bedroom” and (**b**) Test Site II: “The Chamber with a Parrot”. In both Figure 1 and Figure 2, the height of each TLS station related to the reference scan was presented [27].

The TLS data were acquired by the phase scanner Z + F 5003, with the point resolution of 3.2 mm/10 m—one scan with full angular resolution and five scans with a selected fragment of the chamber. Scan positions with distances to the measured wall are presented in Figure 2a.

#### 3.2.2. Test Site II “The Chamber with a Parrot”

**Figure 3:**
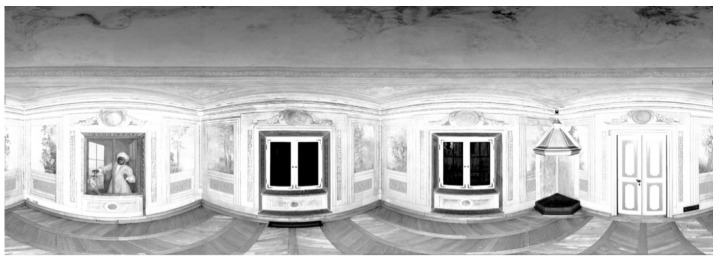
The point cloud in the spherical projection of Test Site II: “The Chamber with a Parrot” without marked points [27].

Test Site II also has a monumental interior, but compared to Test Site I, it has fewer ornaments, and a lack of bas-reliefs, facets, or fabrics on the walls. Despite that, there are spatial effects on the wall, created by wall paintings (Figure 3). For point cloud acquisition, the Z + F 5006 h scanner (its newer generation) was used, and four scans with a horizontal extent of 360° and a scanning resolution of 3.2 mm/10 m were obtained. During the survey, it was not possible to place marked check points.

#### 3.2.3. Test Site III: “The Office”

**Figure 4:**
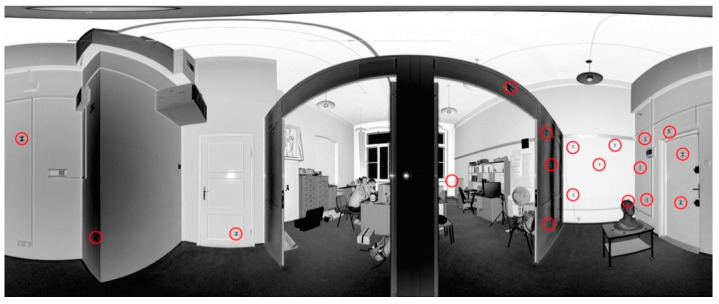
An example of the point cloud in the spherical projection of Test Site III: “The Office” with marked check points (red circles) [27].

**Figure 5:**
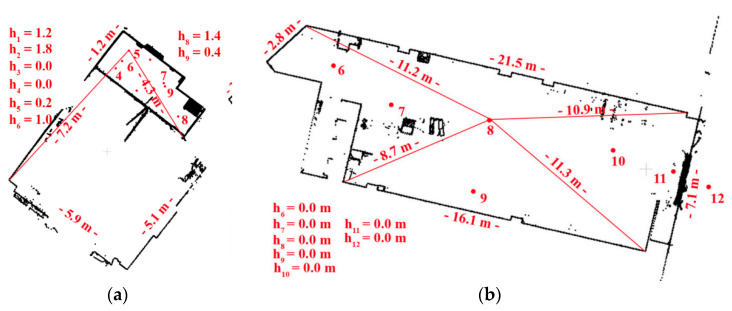
The floor plan with marked TLS scanner position with distances to the nearest walls for: (**a**) Test Site III: “The Office” and (**b**) Test Site IV: “Empty Shop (shopping mall)”. The high point of each TLS station related to the reference scan was presented in figures. The ‘h’ values are related to the high point of the reference scan [27].

Test Site III is an office with a narrow lobby, smooth walls without any texture, and lamps and power wires located on the ceiling. Additionally, the floor was covered with a dark carpet (Figure 4). Figure 5a presents the distribution of eight scan positions (scans were acquired by Z + F 5006 h scanner with a horizontal extent of 360° and a scanning resolution of 6.2 mm/10 m). In order to perform the quality assessment of the TLS data registration, 19 marked check points were used (Figure 4).

#### 3.2.4. Test Site IV: “Empty Shop (Shopping Mall)”

**Figure 6:**
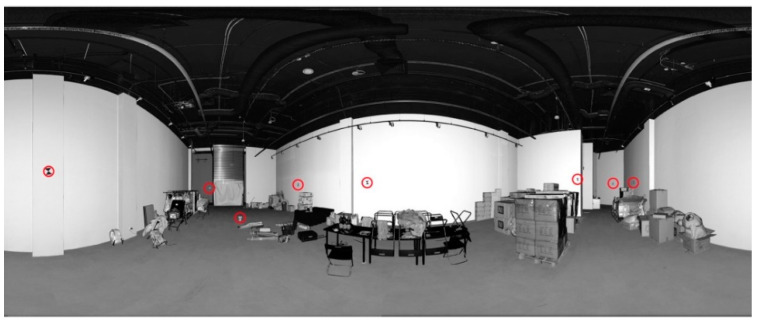
An example of the point cloud in the spherical projection of Test Site IV: “Empty Shop (shopping mall)” with marked check points (red circles) [27].

Test Site IV is an ordinary, empty shop room with smooth walls devoid of any textures, a floor made of concrete with lamps, electric wires, and an air conditioning unit located on the ceiling (Figure 6). In this test site, seven scanners were acquired by Z + F 5006 h scanner with a horizontal extent of 360°, a scanning resolution of 12 mm/10 m and an eight position TLS (scan) was used as a reference (Figure 5b). In Test Site IV, eight marked check points were distributed and used for independent quality assessment.

## 4. Results

In order to assess the selection of the appropriate detector applied in the automated image-based TLS data registration process, the following analyses were performed: (1) time taken for tie points detection and matching, (2) evaluation of the accuracy of automatic matching pairs of scans, (3) accuracy analysis of natural control points, and (4) accuracy analysis of natural check points.

### 4.1. Time Taken for Tie Points Detection and Matching

To assess the impact of the selection of the appropriate point (FAST and BRISK) and blob (ASIFT, CenSurE, and SURF) detector on data registration, processing times were analyzed. Table 1 presents the mean time for detection and matching of the key points of all pairs of raster images in the various projections for all test sites in minutes and seconds, respectively.

When the results presented in Table 1 were analyzed, it was observed that the ASIFT detector had the longest processing time, and the CenSurE detector had the shortest processing time. In general, longer times were recorded for the Mercator projection, the spherical image, and orthoimage, respectively.

### 4.2. Statistics of the Number of Detected and Matched Key Points for Particular Projections of Point Clouds for All Test Sites

In order to assess the effectiveness and accuracy of selected blob and point detectors, the percentage and the number of correctly detected tie points were also analyzed. The process of matching key points was performed in two stages: (1) descriptor matching and (2) geometric matching in the iterative bundle adjustment process. The use of only the descriptor matching step is insufficient because the pairs of key points are not always correctly detected as a result of the matching gradients of changes in grey levels. The incorrect matching of key points, based on the descriptors’ matching results from the distortion of intensity values resulting from TLS measurements, are influenced by the angle and distance of scanning. The use of the bundle adjustment step (with consideration of geometric conditions) enabled the elimination of outliers, resulting from descriptor matching. In order to determine the percentage number of correctly detected tie points, the following equation was used:

(3) $$\frac{the\;final\;number\;tie}{the\;number\;of\;points\;obtained\;by\;the\;key-point\;descriptor\;matching}*100\%$$

Figures 7, 9, 11 and 13 present the mean, maximum, and minimum percentage of correctly detected points, and Figures 8, 10, 12 and 14 present the mean, minimum, and maximum number of detected tie points for correctly oriented pairs of point clouds obtained in the bundle adjustment process.

**Figure 7:**
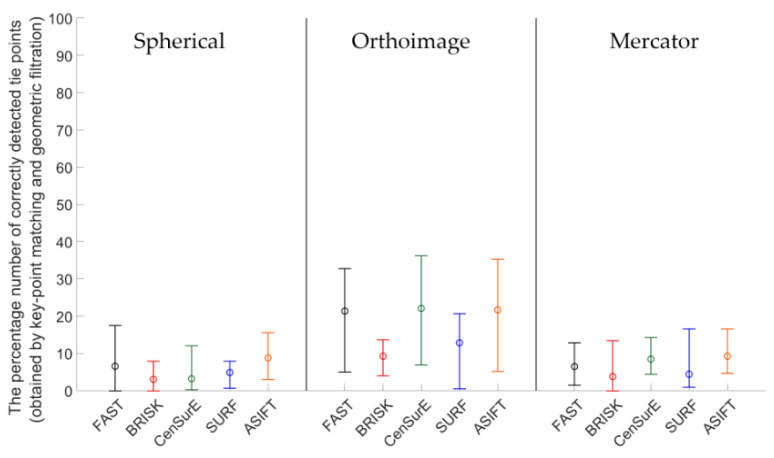
Diagram of the mean (circles), maximum, and minimum percentage of correctly detected and matched key points using blob (ASIFT, SURF, and CenSurE) and point (FAST and BRISK) detectors for rasters in the spherical projection, orthoimages, and the Mercator projection; Test Site I.

Figure 7 presents the percentage of correctly detected and matched key points for point clouds obtained by the Z + F 5003 terrestrial scanner (the first generation Z + F scanner; archival data) and converted into rasters in the spherical projection, orthoimages, and the Mercator projection. The best results (the highest percentage of the number of correctly detected points) were obtained for orthoimages—the values do not exceed 40% for all detectors. In the case of the spherical and Mercator projections, the maximum percentage of correctly detected and matched tie points does not exceed 20%. When the mean values of the percentage of correctly detected tie points were analyzed for all detectors, it was noted that they were similar in the case of rasters in the spherical and Mercator projections. When comparing mean values obtained for the pair of rasters with values obtained for the orthoimages, it was observed that they were approximately four times higher for FAST and CenSurE detectors, and around twice higher for BRISK, SURF, and ASIFT detectors.

**Figure 8:**
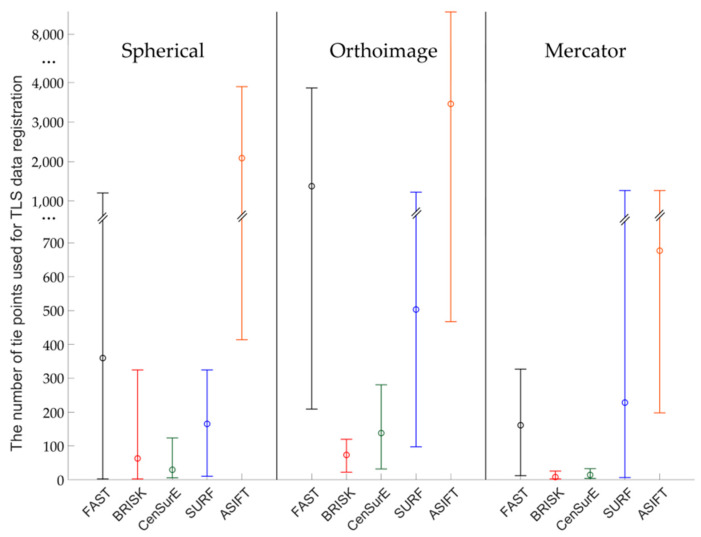
Diagram of the mean (circles), maximum, and minimum number of correctly detected and matched key points using blob (ASIFT, SURF, and CenSurE) and point (FAST and BRISK) detectors for rasters in the spherical projection, orthoimages, and the Mercator projection; Test Site I.

In order to perform a complete assessment of the efficiency of particular detectors for Test Site I, the number of correctly detected tie points should also be compared. An analysis of the maximum, minimum, and mean values presented in Figure 8, identified that the highest number of points were detected using the ASIFT detector for all cartographic projections. Similarly, in the case of the percentage of the number of correctly detected tie points, the best results were obtained for orthoimages. In the case of using rasters in the spherical projection, the number of points detected by means of the ASIFT detector was approximately five times higher, compared to the FAST detector; in the case of the Mercator projection, it was around three times higher, and it was approximately twice higher for the orthoimages. When the diagrams presented in Figure 8 were analyzed, it could be concluded that the smallest number of points for all projections of point clouds was detected using the BRISK and CenSurE detectors, and the highest number of points, for the spherical projection, was detected using the ASIFT, FAST, and SURF detectors, respectively; for the orthoimages, the ASIFT, FAST, and SURF detectors were used and the ASIFT, SURF, and FAST detectors for the Mercator projection.

**Figure 9:**
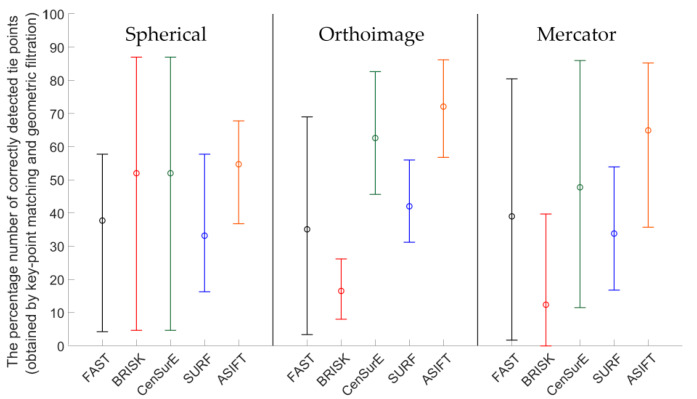
Diagram of the mean (circles), maximum, and minimum percentage of correctly detected and matched key points using blob (ASIFT, SURF, and CenSurE) and point (FAST and BRISK) detectors for rasters in the spherical projection, orthoimages, and the Mercator projection; Test Site II.

The mean, maximum and minimum percentage values of correctly detected and matched key points for Test Site II (Figure 9) are higher than for Test Site I (Figure 7). For all TLS point cloud representations, considerable differences between the results of particular detectors were identified. In the case of the spherical projection, the smallest dispersion of results was obtained for the ASIFT and SURF algorithms, respectively, and the highest distribution was recorded for the BRISK and CenSurE detectors. In the case of orthoimages, the highest percentage efficiency of key point matching was obtained for the ASIFT, CenSurE, and SURF detectors, respectively. The results recorded for the FAST detectors were characterized by high deviations. In the case of using rasters in the Mercator projection, the best results (the mean percentage of correctly detected points) and the smallest values of deviations were obtained for the ASIFT algorithm.

**Figure 10:**
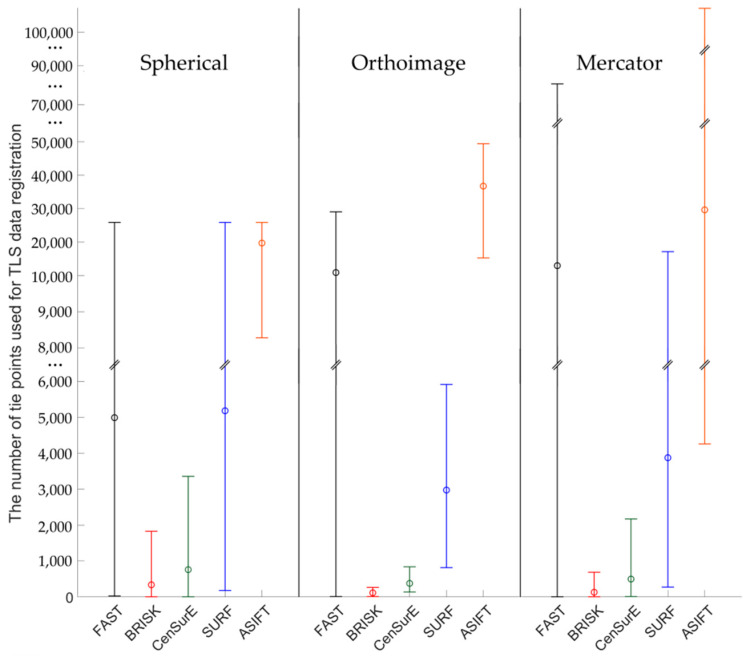
Diagram of the mean (circles), maximum and minimum number of correctly detected and matched key points using blob (ASIFT, SURF, and CenSurE) and point (FAST and BRISK) detectors for rasters in the spherical projection, orthoimages, and the Mercator projection; Test Site II.

The diagrams presented in Figure 10 indicate that the highest, mean number of correctly detected points was recorded by the ASIFT detector. In the case of the spherical projection, a similar, maximum number of detected and matched tie points was detected for the FAST and SURF detectors, the mean values of which were approximately twice smaller than those of the ASIFT detector. In the case of orthoimages, the best results were obtained by the ASIFT and FAST detectors; for rasters in the Mercator projection, the best results were recorded by the ASIFT, FAST, and SURF detectors, respectively. However, considerable distribution of the maximum and minimum values for FAST and ASIFT detectors should be noted. Depending on the assumed projection, maximum values are equal to 27,000, 3000, and 78,000 points in the case of the FAST detector and 27,000, 50,000, and more than 100,000 points for the ASIFT detector for rasters in the spherical projection, orthoimages, and in the Mercator projection. When the results presented in Figure 10 were analyzed, it was observed that a larger mean number of correctly detected and matched tie points was obtained in the case of the CenSurE detector, compared with the BRISK detector. Similar relationships may also be noted in terms of the maximum and minimum values.

**Figure 11:**
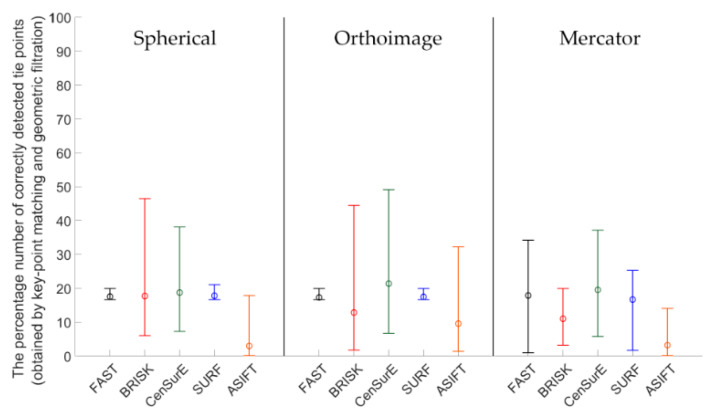
Diagram of the mean (circles), maximum, and minimum percentage of correctly detected and matched key points using blob (ASIFT, SURF, and CenSurE) and point (FAST and BRISK) detectors for rasters in the spherical projection, orthoimages, and the Mercator projection; Test Site III.

Results of the percentage of correctly detected tie points for Test Site III (Figure 11) should be discussed separately for each cartographic projection. For the spherical projection, it may be noted that the mean values of the percentage of correctly detected and matched key points are similar for the FAST, BRISK, SURF, and CenSurE detectors and that they are all within the range of 15–20%; in the case of the ASIFT detector, they are equal to approximately 3%. Considerable differences may be identified in the ranges of the maximum and minimum values; the highest values were obtained for the BRISK detectors and the lower values for the CenSurE and ASIFT detectors. The lowest dispersion of values was obtained for the FAST and SURF detectors. In the case of the orthoimages, similar to the spherical projection, the lowest distribution of values was obtained for the FAST and SURF detectors, while the highest distribution was obtained for the BRISK and CenSurE detectors. Compared to the spherical projection, the higher mean percentage of correctly detected and matched tie points, as well as the higher dispersion value, was recorded for the ASIFT detector. In the case of the Mercator projection, it may be noted that similar mean values were obtained for the FAST, CenSurE and SURF detectors. In the case of the FAST and SURF detectors, a bigger difference between the minimum and the maximum percentage of correctly matched key points was identified than in the case of the spherical projection and orthoimages. Compared to the spherical projection and orthoimages, a smaller percentage of correctly detected tie points was obtained for the ASIFT detector and the Mercator projection.

**Figure 12:**
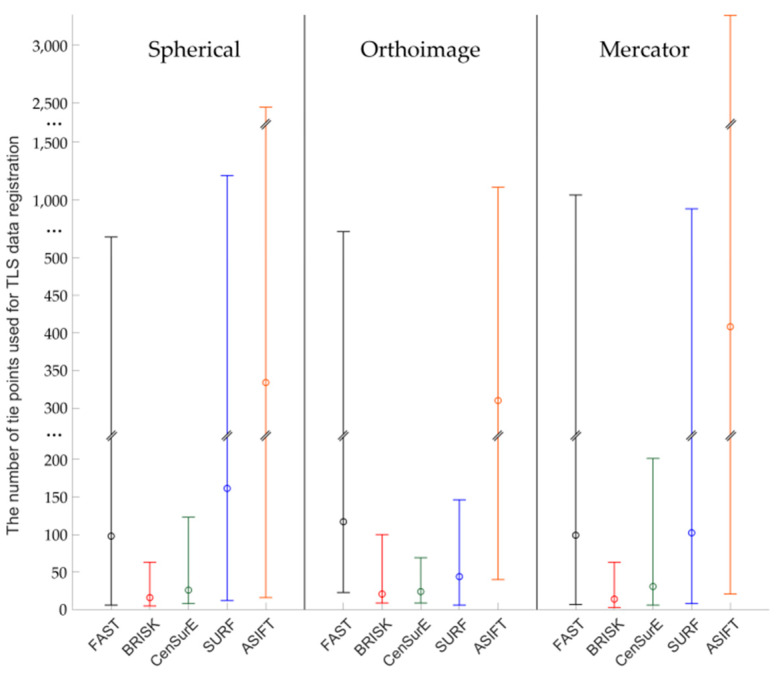
Diagram of the mean (circles), maximum and minimum percentage of correctly detected and matched key points using blob (ASIFT, SURF, and CenSurE) and point (FAST and BRISK) detectors for rasters in the spherical projection, orthoimages, and the Mercator projection; Test Site III.

In the case of Test Site IIII (Figure 12), the highest number of key points was correctly detected and matched by means of the ASIFT detector for all projections, the FAST detector (in orthoimages and rasters in the Mercator projections), and the SURF detector (in the spherical and the Mercator projections), respectively. When the results obtained for the BRISK detector and data obtained from the ASIFT detector were compared, the data indicated that the mean number of detected points was around 20 times smaller for spherical projection, 15 for orthoimages, and 30 for Mercator projection. For the spherical and the Mercator projections, a similar relationship with Test Sites I and II may be observed—the higher mean numbers of points and higher deviations between maximum and minimum values were obtained in the case of the CenSurE detector rather than the BRISK detector.

**Figure 13:**
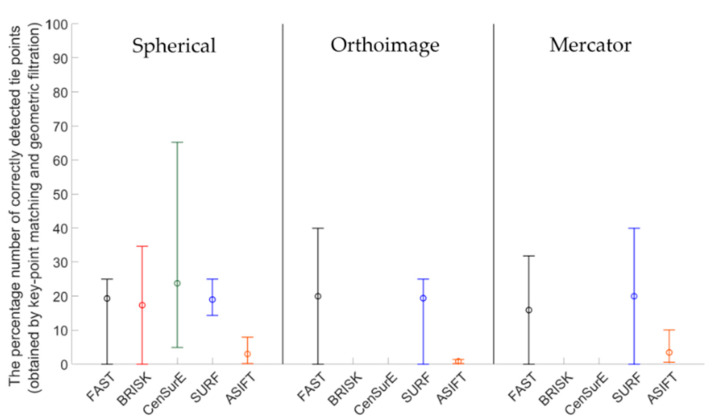
Diagram of the percentage mean (circles), maximum, and minimum number of correctly detected and matched key points using blob (ASIFT, SURF, and CenSurE) and point (FAST and BRISK) detectors for rasters in the spherical projection, orthoimages, and the Mercator projection; Test Site IV.

An analysis of Test Site IV (Figure 13) revealed that the mean values were equal to 0 in the case of the BRISK and CenSurE detectors in orthoimages and rasters in the Mercator projection. This results from the lack of correctly detected tie points. The use of FAST, SURF, and ASIFT detectors allowed for the correct detection of tie points. When comparing the results for the ASIFT, SURF, and FAST detectors, it was noted that the mean percentage of correctly detected tie points was smaller for the ASIFT detector than for the FAST and SURF detectors.

**Figure 14:**
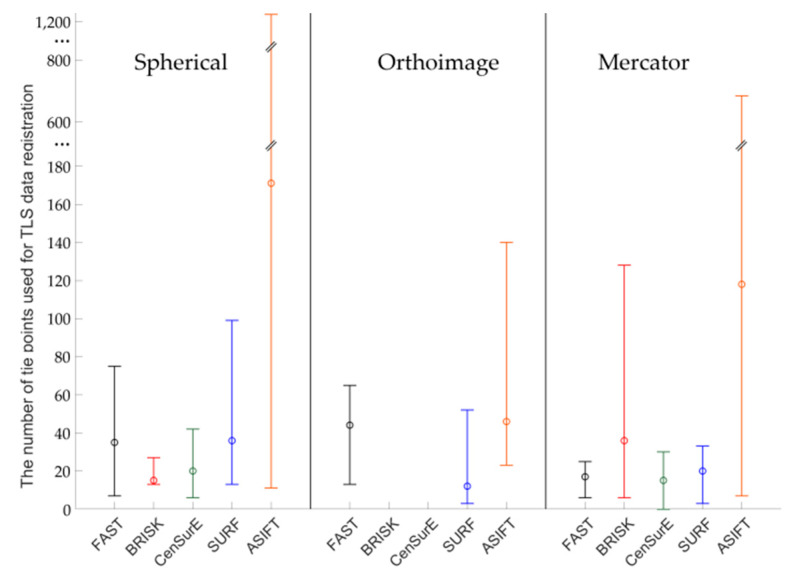
Diagram of the mean (circles), maximum, and minimum percentage of correctly detected and matched key points using blob (ASIFT, SURF, and CenSurE) and point (FAST and BRISK) detectors for rasters in the spherical projection, orthoimages, and the Mercator projection; Test Site IV.

The results presented in Figure 14 indicate that the mean and maximum values of correctly detected tie points were obtained for the ASIFT algorithm (around 45 times more than BRISK and spherical projection, 2 times FAST and orthoimages, 35 times more than FAST and Mercator projection for maximum values). When the other values presented in Figure 14 were analysed, it was noted that it was not possible to correctly detect and match key points, used in further stages of TLS point cloud registration by means of BRISK and CenSurE detectors for orthoimages. In the case of the spherical projection, the mean values of the number of points for FAST and SURF detectors were similar, equal to approximately 40 points; the maximum values were equal to around 75 points for the FAST detector and approximately 100 points for the SURF detector. In the case of the BRISK and CenSurE detectors, the mean values were smaller and equal to around 20 points. In the case of orthoimages, the FAST and ASIFT detectors allowed for the correct detection of approximately 45 tie points and the SURF detector, around 15 points on average. Considerable differences (around two times) were observed for the maximum values and between ASIFT and FAST/SURF detectors. In the case of the Mercator projection, the FAST, CenSurE, and SURF detectors enabled the detection of approximately 20 tie points on average, and the BRISK detector allowed the detection of around 40 points. The maximum and minimum values for the CenSurE and SURF detectors were similar; the maximum value for the BRISK detector was approximately six times higher.

### 4.3. Evaluation of the Accuracy of Automatic Matching of Pairs of Scans

To assess the effectiveness of the detectors in the image-based TLS data registration process, not only the analysis of the number of tie points is required, the accuracy of the orientation of pairs of scans acquired from different heights and distances from scanned surfaces, should also be considered. Table 2, Table 3, Table 4 and Table 5 present the results obtained from different test sites and different projections (Test Sites I–IV). The results are marked in color: (1) full registration (green), where the RMSE of the points in coordinates X, Y, and Z ≤ 0.02 m (for Test Sites I–III)/≤ 0.04 m (for Test Site IV), and points are evenly distributed within the analyzed area, (2) preliminary orientation parameters that should be used in the ICP (orange), and (3) no registration (red). The symbol “x” indicates that pairs of scans could not be connected due to insufficient overlap.

Regardless of the selected projection and the pair of scans, the best results were obtained for the ASIFT detector (Table 2). The use of orthoimages enables a high efficiency of processing (that should be understood as the correctness of the pair of scan registration—number of correct registered TLS data (full registration) without additional processing, i.e., ICP method) to be obtained, as is also the case for the point (FAST and BRISK) and blob (CenSurE, SURF, and ASIFT) detectors. Only in the case of the FAST (two pairs of scans) and BRISK (one pair of scans) detectors and the spherical projection was it not possible to perform the full registration and to determine approximate parameters for data orientation by means of the ICP method. The use of the Mercator projection allowed the elimination of the problem of orientation of the above pairs of point clouds for FAST and BRISK algorithms. However, the use of the Mercator projection and the BRISK, CenSurE, and SURF algorithms resulted in the lowering of the accuracy of the data orientation process, which rendered an additional orientation, using the ICP method to be carried out.

Similar to Test Site I (Table 2), the best results for Test Site II (Table 3) were obtained using the ASIFT algorithm, regardless of the type of the assumed projection. However, the full effectiveness in such a case (full registration, 100%) was also obtained in the case of the SURF and FAST detectors. Similar to Test Site I, the use of orthoimages for the CenSurE detector enabled an improvement of the TLS data registration process, without requiring a final orientation using the ICP method to be performed. The use of points detected by means of the BRISK detector in orthoimages allowed full registration to be carried out (for the pair of scans 5 and 6); in the case of the pair of scans 4 and 5, it resulted in lowering the accuracy of the data registration process and forced the final registration using the ICP method. Similar to Test Site I, the use of the Mercator projection contributed to lowering the accuracy of the orientation process with the use of points detected, by means of the BRISK and CenSurE detectors.

The effectiveness of particular algorithms was not only evaluated in the cases of test sites connected with surveys of cultural heritage objects. Using solely monumental surfaces for the analysis, testing point and blob detectors would be unreliable, since such objects are usually highly diversified with respect to architectural details, colors, and types of surfaces. This transforms into a high diversification of gradients of grey level changes, which greatly influence the number of correctly and evenly detected and matched tie points. Therefore, the authors decided to analyze the effectiveness of the operations of the above detectors using two additional test sites: Test Site III (Table 4), which is an office with minimal texture and contrast of analyzed surfaces and without any architectural detail, and Test Site IV (Table 5), an empty shop in a shopping mall, with neither advertisements nor architectural details and with smooth, white walls.

The point clouds for Test Site III were acquired with a full angular resolution (360°/310°) and the TLS positions did not guarantee beneficial geometric conditions because of their location close to the walls (Figure 7). Similar to the previous test sites (Table 2 and Table 3), all point clouds (regardless of the assumed type of cartographic projection) were correctly oriented using points detected by the ASIFT detector. The use of orthoimages allowed the detection of tie points for the FAST (all pairs of point clouds were correctly oriented), CenSurE (only two of the pairs of scans were correctly oriented), and BRISK detectors. When the results from the matching point clouds using the SURF detector were compared with the orthoimages and spherical images, a worsening of the results could be observed. The use of the TLS point cloud conversion in the Mercator projection with the FAST, CenSurE, and SURF detectors resulted in the lowering of the accuracy of point cloud orientation; only the combination with the BRISK detector enabled a slight improvement of the registration (an increased number of pairs with full registration and ICP).

When the operations of particular detectors were analyzed for Test Site IV (Table 5), it was observed that only in the case of spherical projection was it possible to perform full registration of all pairs of scans for FAST, CenSurE, and ASIFT detectors; in the case of the Mercator projection, it was only possible for the ASIFT detector. For such types of analyzed objects, it was not recommended to use orthoimages in the point cloud orientation process or rasters in the Mercator projection.

### 4.4. Accuracy Analysis of Natural Control Points

**Figure 15:**
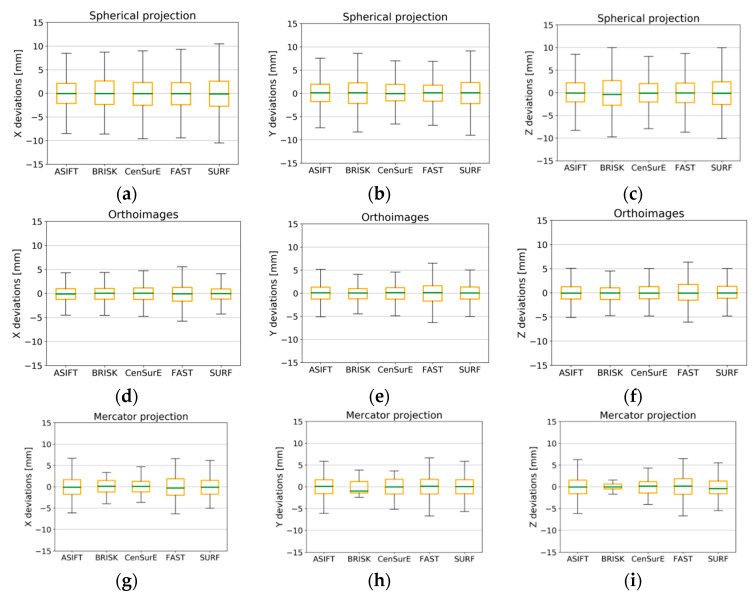
Box plots for the distribution of the deviations of natural control points for all pairs of fully registered scans (without points, sufficient only for the ICP, or unsuitable for registration) for Test Site I. The spherical projection: component—x (**a**), y (**b**), and z (**c**); orthoimages: component—x (**d**), y (**e**), and z (**f**); the Mercator projection: component—x (**g**), y (**h**), and z (**i**).

**Figure 16:**
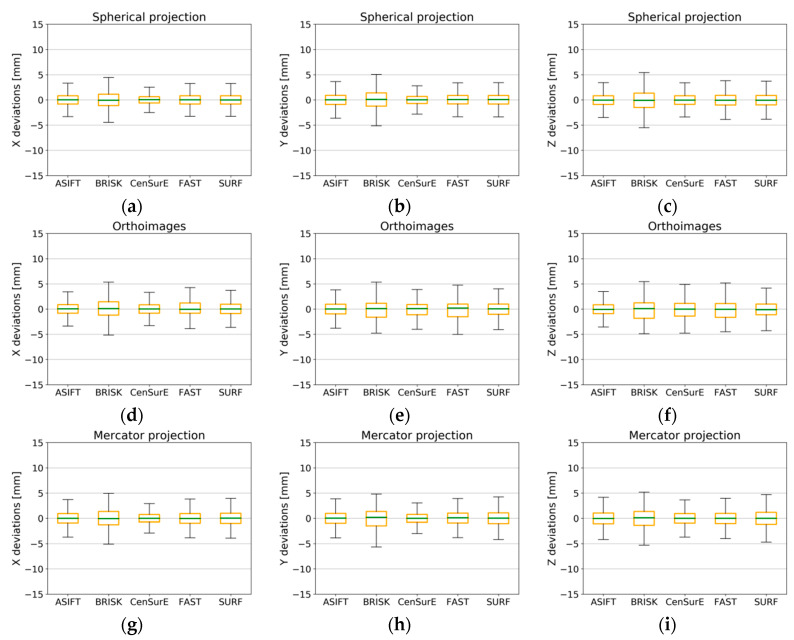
Box plots for the distribution of deviations of natural control points for all pairs of fully registered scans (without points used for the ICP and non-registration) for Test Site II. The spherical projection: component—x (**a**), y (**b**), and z (**c**); orthoimages: component—x (**d**), y (**e**), and z (**f**); the Mercator projection: component—x (**g**), y (**h**), and z (**i**).

**Figure 17:**
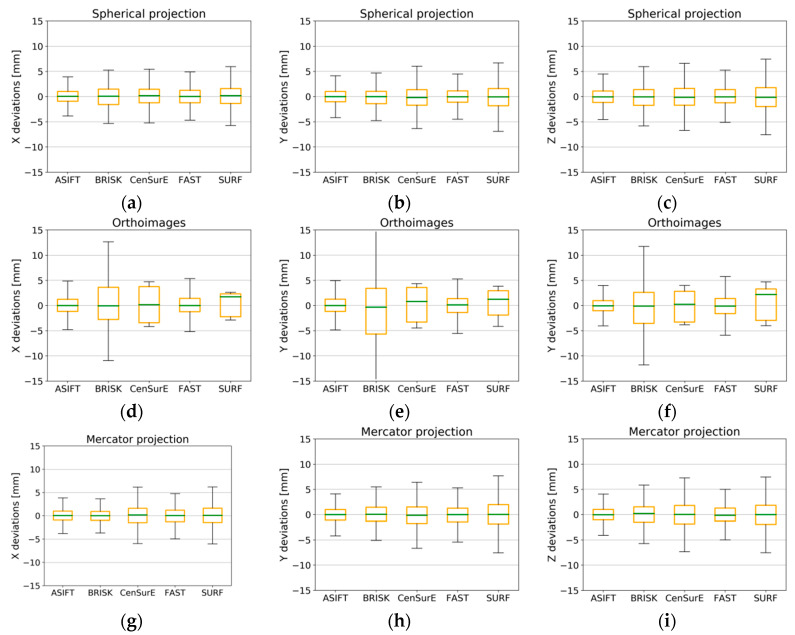
Box plots for the distribution of the deviations of natural control points for all pairs of fully registered scans (without points used for the ICP and non-registration) for Test Site III. The spherical projection: component—x (**a**), y (**b**), and z (**c**); orthoimages: component—x (**d**), y (**e**), and z (**f**); the Mercator projection: component—x (**g**), y (**h**), and z (**i**).

**Figure 18:**
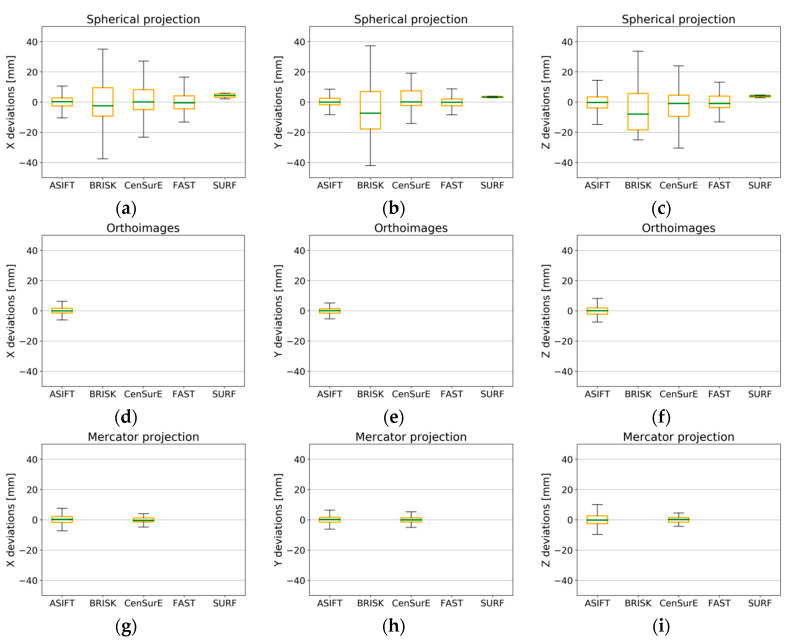
Box plots for the distribution of the deviations of natural control points for all pairs of fully registered scans (without points used for the ICP and non-registration) for Test Site IV. The spherical projection: component—x (**a**), y (**b**), and z (**c**); orthoimages: component—x (**d**), y (**e**), and z (**f**); the Mercator projection: component—x (**g**), y (**h**), and z (**i**).

In order to assess the accuracy of the point cloud orientation process, values of deviations (from the full registration) of automatically detected tie points were divided into the control points used; those points were used to build a mathematical model and to determine parameters of orientation and check points, used for independent quality assessment. In order to assess the TLS data registration based on different detectors, boxplots were chosen for the deviation analysis (Figure 15, Figure 16, Figure 17 and Figure 18).

When the results presented in Figure 15a,b (the spherical projection) were analyzed, it was observed that the deviations of the natural control points did not exceed ±10 mm, and between the first quartile (Q_1_) and the third quartile (Q_3_), the deviations of points were smaller than ±2 mm (orange box). The median for all cases (the green line) was approximately equal to 0 mm, which proves the lack of systematic errors, and the maximum and minimum values for certain detectors were smaller than ±2 mm.

In the case of orthoimages (Figure 15d–f), deviations were recorded at the lowest values, and the values of the first and the third quartiles for the ASIFT, BRISK, and CenSurE detectors were close to the values obtained based on the points from the spherical and the Mercator projections. In the case of the FAST detector the maximum and minimum values, and the Q_1_ and Q_3_ were approximately ±1 mm greater than the values obtained for the other detectors.

In the case of the Mercator projection (Figure 15g–i), BRISK detector values were considerably different with respect to the maximum and minimum values and the Q_1_/Q_3_. The median values (the green line) for the BRISK (Figure 15h, y-component) and SURF (Figure 15i, z-component) detectors were not equal to zero in this case, and they oscillated within the range of ±1 mm. Other detectors were equal to 0, which proves the lack of gross errors in the observations. The values of deviations between the Q_1_ and Q_3_ did not exceed ±1 mm for the ASIFT, FAST, and SURF detectors, and the maximum and minimum values fell within the range of ±7.5 mm. The use of the BRISK detector enabled the lowest values of deviations to be obtained.

When analyzing the results presented in Figure 16 (for point clouds in all cartographic projections), it was observed that the deviations of natural control points did not exceed ±5 mm in the majority of cases, and they fell within the range of ±1 mm within the Q_1_ and Q_3_. In all cases, the median was equal to approximately 0. When the results obtained for Test Site II were compared with the results obtained for Test Site I, the distribution of the maximum and minimum values of deviations were identified as being two times smaller, and they were similar for the ASIFT, CenSurE, FAST, and SURF detectors. The BRISK detector was characterized by a higher distribution of the values of deviations.

In the case of results for Test Site III (Figure 17) for the spherical and the Mercator projections, there was a similar distribution of the values of deviations of natural control points. The maximum and minimum values for the ASIFT, BRISK, and FAST detectors did not exceed ±5 mm, and in the case of the CenSurE and SURF detectors, they did not exceed ±7 mm. The best results (the smallest maximum and minimum values, as well as values of the Q_1_ and the Q_3_) were obtained for the ASIFT detector for all projections. Other detectors were characterized by a similar accuracy of ±2 mm in the case of rasters and in the spherical and Mercator projections. The highest accuracy of orientation of natural control points was noticeable for the orthoimages and the ASIFT and FAST detectors, for which the median value equaled 0 mm. The median value was not equal to 0 mm in the case of the BRISK, CenSurE, and SURF detectors, which proves a lower accuracy of TLS data registration and the probable occurrence of systematic errors. The maximum and minimum values of deviations for points detected by means of the BRISK detector did not exceed ±10 mm, and the values of the Q_1_ and the Q_3_ fell within the range of ±4 mm for the X, Y, and Z coordinates for the BRISK and CenSurE detectors, for components X and Y for the SURF detector, and ±4.5 mm for component Z.

The results obtained for Test Site IV (Figure 18) were highly diversified and incomparable with the results of previous test sites. The boxplot does not exist in the diagrams of orthoimages and the Mercator projection (Figure 18d–i); this results from a lack of detection of correct tie points by certain detectors. When the results for the spherical projection were analyzed (Figure 18a–c), it was observed that the best results were obtained for the ASIFT detector and for the CenSurE, BRISK, and FAST detectors, respectively. In the case of the SURF detector, a systematic shift of the median by approximately 5 mm was noted. When the results obtained for Test Site IV were compared with the results of other test sites, the obtained values showing the deviations for raster in the spherical projection and for specified detectors were considerably different, and the maximum and minimum values exceeded ±10 mm. The best results were obtained for the ASIFT detector for all projections, and the use of orthoimages and rasters in the Mercator projection enabled accuracy values comparable with the results recorded for Test Site I to be obtained (the monumental test site which is characterized by a high number of architectural details) and for Test Site III (the office).

### 4.5. Accuracy Analysis of Natural Check Points

A similar accuracy analysis was performed on natural check points which are not used to build the photogrammetric model or determine elements of orientation of point clouds but are used to independently check the results obtained.

**Figure 19:**
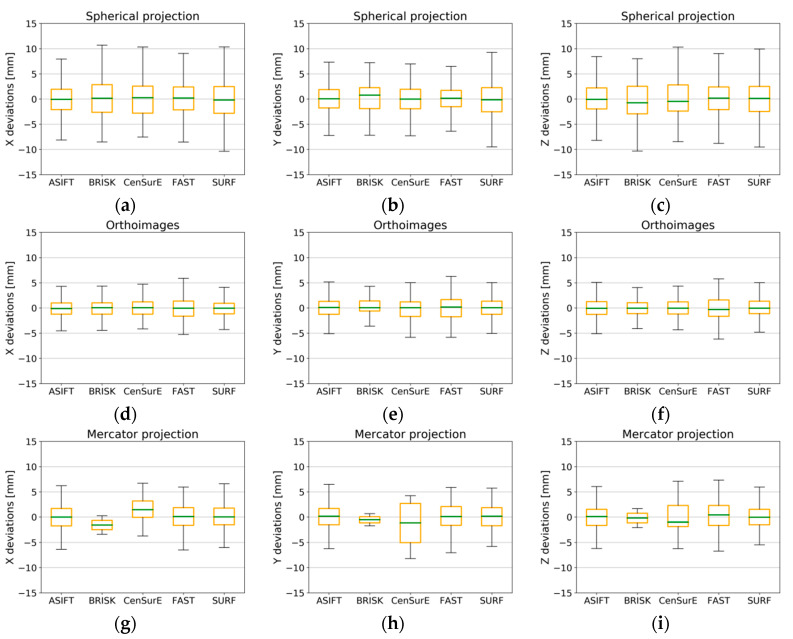
Box plots for the distribution of the deviations of natural check points for all pairs of fully registered scans (without points used for the ICP and non-registration) for Test Site I. The spherical projection: component—x (**a**), y (**b**), and z (**c**); orthoimages: component—x (**d**), y (**e**), and z (**f**); the Mercator projection: component—x (**g**), y (**h**), and z (**i**).

The distribution of deviation values of natural check points for Test Site I (Figure 19) is similar to the values of deviations of natural control points, which proves the correct detection and matching of tie points, and the correct performance of TLS data registration. For rasters in the spherical projection for ASIFT and CenSurE detectors, the maximum and minimum values of deviations did not exceed ±10 mm, and these values did not exceed approximately ±11 mm in the case of other detectors. All detectors recorded similar values in the interval between the Q_1_ and the Q_3_; those values were mutually different by around ±1 mm only. The maximum and minimum values of deviations for points detected in orthoimages for all detectors did not exceed ±6 mm, values of the Q_1_ and the Q_3_ were similar, and the median was equal to 0 mm. This proves the high accuracy of TLS data registration. The highest differences in the values of deviations were obtained in the case of points detected in rasters in the Mercator projection. For BRISK and FAST detectors, the non-zero value of the median was noted, which could prove the existence of systematic errors in the TLS point cloud registration process. The maximum and minimum values of deviations for rasters in the Mercator projection did not exceed ±7 mm. In the case of the median, the differences could easily be identified. The values of the median for the ASIFT, CenSurE, and SURF detectors were equal to 0 mm, and for the remaining detectors, they differed from 0 by approximately ±1 mm. The values of the Q_1_ and the Q_3_ for the FAST detector were smaller than those of other detectors; the BRISK detector recorded the highest values

**Figure 20:**
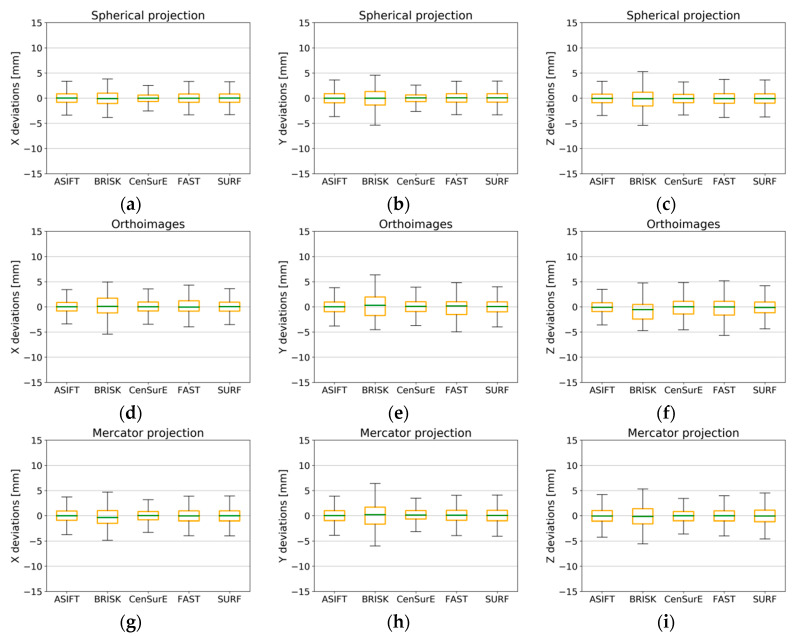
Box plots for the distribution of the deviations of natural check points for all pairs of fully registered scans (without points used for the ICP and non-registration) for Test Site II. The spherical projection: component—x (**a**), y (**b**), and z (**c**); orthoimages: component—x (**d**), y (**e**), and z (**f**); the Mercator projection: component—x (**g**), y (**h**), and z (**i**).

The deviations of the natural check points and natural control points for Test Site II (Figure 20) are similar, which proves the correct detection and matching of tie points, as well as the accurate adjustment of observations, as in the case of Test Site I. Only in the case of the BRISK detector (for all projections of point clouds) did the maximum and minimum values exceed ±5 mm; with the other detectors they were less than ±5mm. The values of the first and third quartiles for the BRISK detector were within the range of ±2 mm on average; other detectors were also within the range of ±2 mm.

**Figure 21:**
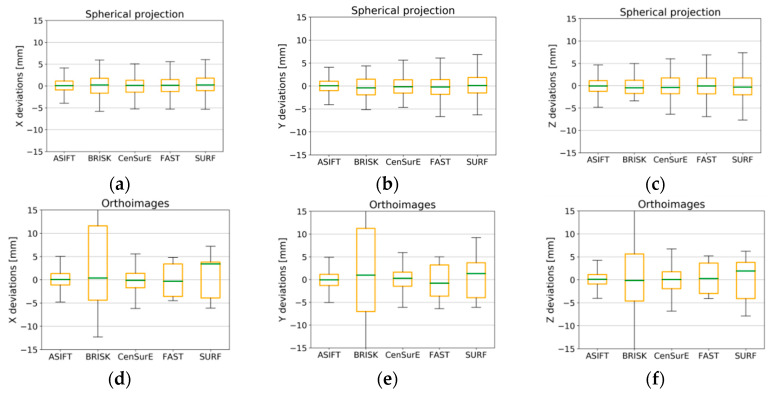

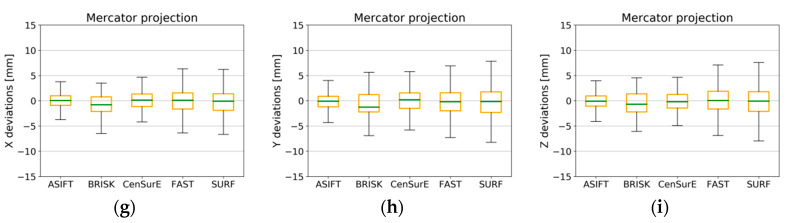
Box plots for the distribution of the deviations of natural check points for all pairs of fully registered scans (without points used for the ICP and non-registration) for Test Site III. The spherical projection: component—x (**a**), y (**b**), and z (**c**); orthoimages: component—x (**d**), y (**e**), and z (**f**); the Mercator projection: component—x (**g**), y (**h**), and z (**i**).

The distribution of the deviation values of the natural check points for Test Site III is similar to the distribution of values of natural control points, acquired through the use of all detectors and rasters in the spherical and the Mercator projections. The use of the ASIFT detector and its cartographic projections recorded the highest accuracy, in which the maximum and minimum value of deviations did not exceed ±5 mm and the values between the first and the third quartiles were within the range of ±1 mm. The use of the BRISK detector with orthoimages for Test Site III resulted in the maximum and minimum values of deviations exceeding ±15 mm; the values between the first and the third quartiles for components X (Figure 21d) and Y (Figure 21e) were within the range of -6 to 12 mm, and component Z was within the range of ±5 mm.

**Figure 22:**
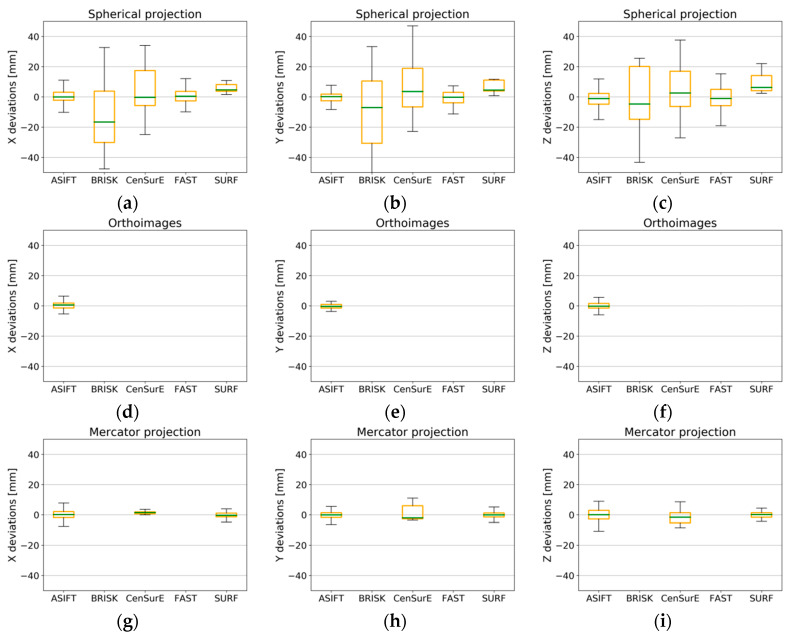
Box plots for the distribution of the deviations of natural check points for all pairs of fully registered scans (without points used for the ICP and non-registration) for Test Site IV. The spherical projection: component—x (**a**), y (**b**), and z (**c**); orthoimages: component—x (**d**), y (**e**), and z (**f**); the Mercator projection: component—x (**g**), y (**h**), and z (**i**).

An analysis of data for Test Site IV, presented in Figure 22, demonstrated that it was only possible for the ASIFT detector to perform the TLS data registration process (deviations smaller than ±15 mm) with the use of all cartographic projections. The highest values of registration accuracy were obtained in this case for the orthoimages (Figure 22d–f). In the case of the spherical projection and other detectors, it was noted that only in the case of the FAST and ASIFT algorithms was the median equal to 0, which proves the lack of gross errors. Besides, the high accuracy of TLS data orientation was also proved by the error value, which fell within the range of ±15 mm for the maximum and minimum values. For other detectors (BRISK, CenSurE, and FAST), the maximum deviations exceeded 20 mm (double the value of the assumed scanning resolution) which is unacceptable.

## 5. Discussion

The article [27] presents the possibilities and limitations of the aforementioned methods, using the commonly applied blob (SURF) and point (FAST) detectors. The authors of this paper decided to extend the scope of the detectors reviewed by including ASIFT (the detector which considers the influence of affinity), BRISK (the extended version of the FAST algorithm, based on image pyramids), and CenSurE (center–surround filters, used for real-time calculations and decreasing calculation time). Moreover, the key factors which influence the selection of the optimum detector were also analyzed. They include: (1) the time for searching and matching tie points, (2) the percentage and number of correctly detected tie points, and (3) the values of deviations of natural and automatically detected control and check points on rasters in the spherical projection, the Mercator projection, and in orthoimages.

The first analyzed factor which influences the selection of the optimum detector in the TLS data registration process is the time required to search for and match key points. When the results presented in Table 1 are analyzed, it may be noted that, on average, the ASIFT detector had the longest processing time (even 100 times longer) and the CenSurE, as well as the BRISK, FAST, and SURF detectors, respectively, had the shortest time across all projections and all test sites. However, consideration should be given not only to the time taken to search and match key points; the number of correctly detected points should be also taken into account. The proposed approach to determine the tie points was a two-stage approach. During the first stage, descriptor matching was performed, and during the second stage, possible pairs of points were filtered based on geometric relations and the RANSAC algorithm. The use of such an approach resulted from the specific features of utilized source data. It was assumed that the aforementioned detectors and the descriptor, were created to search for characteristic points in 2D images. Images that are the results of 3D point cloud conversion into the raster 2D form (using different cartographic transformations) are characterized by the presence of distortion, which does not occur in centrally projected images. Besides, as the intensity of laser beam reflectance differs and depends on the distance and the scanning angle, similar surfaces are characterized by the different intensity. Those two features influence the necessity to perform the additional filtration, which considers geometric relations between detected and matched points according to the descriptor matching procedure. The highest percentage of correctly matched key points was obtained in the case of the BRISK detector; the smallest number was obtained for the ASIFT detector. However, reverse results were obtained with regard to the number of points. It is possible to correctly select the detector in image-based registration, due to the accuracy analysis of orientation and the number of correctly oriented pairs of scans. The analyses carried out indicated that different numbers of key points are detected using different descriptors. Therefore, attention should not be focused on the percentage of correctly detected and matched key points only. The mean number of points detected by the ASIFT detector is five times higher than the number of points detected by other algorithms. When the detector is selected for image-based TLS registration, consideration should be given as to whether it is important to ensure a shorter data processing time and a smaller number of correctly detected tie points, or to obtain a greater number of tie points within the longer time of data processing. In the case of processing cultural heritage objects, the use of an ASIFT detector (which is characterized by a data processing time of around six times longer than other detectors) is not necessary, which enables more tie points to be detected; instead, it is recommended to use other detectors or combinations, such as FAST and CensSurE detectors which allow a similar number of points, characterized by a lower computational complexity, to be obtained. In the case of public utilities, the use of the ASIFT detector enables a 100% full registration to be carried out, and the selection of other detectors is associated with the supervision of data analysis and, in some cases, with the final registration of the ICP method.

The target-based method, based on marked control points, is the commonly applied method of TLS data orientation. Those marked control points must be evenly distributed across the processed object. Another approach is the ICP method which automatically orientates scans “cloud-to-cloud” in the condition of their initial orientation. In the case of cultural heritage objects, due to their nature, it is sometimes not possible to distribute marked points on an object, and the location of control points on surveying tripods does not ensure the required geometric properties. Therefore, it is recommended to apply the image-based TLS registration and the selection of the detector depends on the processing time. In the case of processing objects of other types (such as offices or shopping malls), although it is possible to place control points on the object, it is not always possible to evenly distribute those points (often due to physical limitations, such as heights of rooms, etc.) and to ensure the correct geometry of the control points. In such cases, it is reasonable to apply the image-based TLS data registration method, which is not based on the marked points of the control. For a reliable assessment of the accuracy analysis performed, marked points of the control were used as reference points. They were measured, using the software application dedicated for the Z + F LaserControl scanner, using the target-based method.

The accuracy of target-based method is determined by two factors that should be considered: the measured range error (that for Z + F 5003 is <6 mm and Z + F 5006 h is <1 mm) and distance between TLS and targets/walls that affect the point cloud density. The following parameters were determined for each test site (1) Test Site I—distances: 4–5 m (Figure 2a); the point cloud resolution: approximately 2 mm; (2) Test Site III—distance: approximately 4 m (Figure 5a), which affects 3 mm point cloud resolution; and (3) Test Site IV—distance: approximately 11 m (Figure 5b), point cloud resolution: 12 mm. When the orientations performed using the image-based and the target-based method were compared (Table 6), evidence showed that in the case of archival point clouds (acquired by the first generation Z + F scanner, Test Site I), the accuracy of registration (on spherical images) with the use of detectors was always higher than the accuracy of the commonly applied target-based method. The best results were obtained for the ASIFT detector and the worst results for the BRISK detector. It should be stressed that in the case of such types of objects, it is recommended to use rasters in the spherical projection or in the Mercator projection, because registration accuracy (for FAST, CenSurE, SURF, and ASIFT) is higher than range accuracy. The accuracy of operations, performed by detectors, is considerably lower for “office” spaces (Test Site III), which are not characterized by a high degree of detail. Only by using the ASIFT detector and orthoimages was the satisfaction of achieving a similar registration accuracy obtained, an accuracy higher than scanning resolution. However, it should be stressed that the values of accuracy recorded, still allow for correct data registration, since the obtained values of accuracy for the process are lower than the assumed scanning resolution. In the case of surfaces which do not have a diversified texture and which are characterized by high gradients of change in grey level values, the best results were obtained for the FAST detector (the point detector) with raster in spherical projections and for the ASIFT detector (the blob detector which considers affinity) with raster in all projections. The results obtained for Test Site IV (an empty shop in a shopping mall) for all detectors with the exception of the ASIFT detector should be considered only as an approximation of the final orientation process, using the ICP method. The results obtained for the ASIFT detector for the spherical projection and the Mercator projection are similar to the assumed scanning resolution; therefore, they may be considered accurate, although they are around three times worse than the results obtained in the target-based method.

When the decision to select the detector in the image-based TLS registration process is made, not only the total and the percentage of detected tie points or acquired RMSE values should be considered; attention should also be paid to the percentage effectiveness of connecting pairs of point clouds. Table 2, Table 3, Table 4 and Table 5 indicated that only the use of the ASIFT detector and the point cloud converted to the raster form in the spherical projection, and in the Mercator projection, it enabled the full registration of TLS point clouds. In the case of the BRISK and CenSurE detectors, the use of orthoimages increased the accuracy of the orientation process (Test Site I and II) compared to other projections. When objects of a poor texture were processed (Test Site III and IV), the use of the ASIFT detector enabled a 100% orientation of all point clouds from terrestrial laser scanning, and the use of appropriate FAST and SURF detectors allowed for the orientation of selected, commonly overlapping point clouds to be performed. BRISK and CenSurE detectors are characterized by lower stability, and therefore, additional registration using the ICP method may be required.

## 6. Conclusions

The research has focused on the impact of the selection of a detector, on account of the quality of the process of automatic image-based TLS data registration, based on point clouds converted into the spherical projection, the Mercator projection, and orthoimages. The experiments performed were carried out in two types of test sites, located within cultural heritage buildings and public utilities, which allowed for the independent analysis of data, characterized by different numbers of architectural details and colors, as well as complicated geometric features. The selection of an appropriate detector and the raster form of the point cloud enables the best completeness in terms of processing time and the highest accuracy of the TLS data orientation process. A summary of the evaluation of the usefulness criteria of point detection algorithms for all test sites on a scale from 1 to 5 is shown in Table 7.

The analysis has shown that the ASIFT algorithm enabled accuracy values to be obtained, which are similar to the values recorded from use of the target-based method and lower accuracy values than the scanning resolution and the completeness of the data registration process for all test sites, using the spherical projection and the Mercator projection. Despite these advantages, that algorithm is considerably slower than other algorithms (BRISK, FAST, SURF, and CenSurE). Therefore, when the orientation of scans starts, the selection criteria of other potentially appropriate detectors should also be considered.

Cultural heritage objects are often characterized by surfaces of diversified shapes, colors, and architectural details. Therefore, it is generally possible to use all types of point and blob detectors. Due to the potentially lower accuracy which can result, in some cases, the points detected using BRISK and CenSurE detectors may be used to determine parameters of the initial orientation for the ICP method. It may be observed that the use of orthoimages allows for more tie points to be detected and increases the registration accuracy. It should also be noted that data from a different generation of the Z + F scanner were used for analyses and that the proposed method of orientation is independent from the equipment applied and has enabled results to be obtained which are close to those recorded using the target-based method.

In the case of interiors of the “public utilities” type, which are geometrically uncomplicated, with uniform colors, the best results of TLS data orientation were obtained in the case of the ASIFT detector in the Mercator projection or in the spherical projection. Such affine detectors are successfully applied in image-based TLS data registration. As regards the remainder of the detectors, attention should be paid to the method of distribution of the scanner stations and the selection of an appropriate representation of point clouds in a raster form (i.e., in the spherical projection, in the Mercator projection, or in the form of orthoimages), since this influences the accuracy and completeness of the automated TLS data orientation process.

The results obtained proved that the ASIFT detector recorded the best results. Therefore, the authors plan to analyze the remaining detectors, considering the impact of affinity.

## Figures and Tables

**Table 1:** Time taken for detection and matching of characteristic points for particular projections of point clouds, for all test sites.

**Detector**	**Average Computation Time [Seconds]**					
	**Test Site I**			**Test Site II**		
	**Cartographic Transformation**					
	**Spherical**	**Orthoimage**	**Mercator**	**Spherical**	**Orthoimage**	**Mercator**
FAST	17	60	106	242	61	211
BRISK	41	22	28	43	32	16
CenSurE	26	8	24	18	12	179
SURF	435	14	250	537	125	370
ASIFT	1807	405	2428	1942	303	2211
**Detector**	**Average Computation Time [Seconds]**					
	**Test Site III**			**Test Site IV**		
	**Cartographic Transformation**					
	**Spherical**	**Orthoimage**	**Mercator**	**Spherical**	**Orthoimage**	**Mercator**
FAST	13	4	104	242	61	211
BRISK	15	12	14	14	28	23
CenSurE	20	6	17	13	16	31
SURF	43	14	250	537	125	370
ASIFT	2592	446	1942	1420	375	2126

**Table 2:** The accuracy of the automated registration of scans for Test Site I (both scenarios): green, correct matching (full registration); orange, preliminary orientation requiring final registration using the Iterative Closest Point (ICP) method; and red, no matching. “x” indicates no connection between scans (no overlap).

**Test Site I—Spherical Projection**																													
Scan no.	FAST					Scan no.	BRISK					Scan no.	CenSurE					Scan no.	SURF					Scan no.	ASIFT				
	3	6	8	9	19		3	6	8	9	19		3	6	8	9	19		3	6	8	9	19		3	6	8	9	19
1		X	X	X		1		X	X	X		1		X	X	X		1		X	X	X		1		X	X	X	
3		X		X		3		X		X		3		X		X		3		X		X		3		X		X	
6					X	6					X	6					X	6					X	6					X
8						8						8						8					X	8					
9						9						9																	
**Test Site I—Orthoimages**																													
Scan no.	FAST					Scan no	BRISK					Scan no.	CenSurE					Scan no.	SURF					Scan no.	ASIFT				
	3	6	8	9	19		3	6	8	9	19		3	6	8	9	19		3	6	8	9	19		3	6	8	9	19
1		X	X	X		1		X	X	X		1		X	X	X		1		X	X	X		1		X	X	X	
3		X		X		3		X		X		3		X		X		3		X		X		3		X		X	
6					X	6					X	6					X	6					X	6					X
8						8						8						8						8					
9						9						9						9						9					
**Test Site I—Mercator Projection**																													
Scan no.	FAST					Scan no	BRISK					Scan no.	CenSurE					Scan no.	SURF					Scan no.	ASIFT				
	3	6	8	9	19		3	6	8	9	19		3	6	8	9	19		3	6	8	9	19		3	6	8	9	19
1		X	X	X		1		X	X	X		1		X	X	X		1		X	X	X		1		X	X	X	
3		X		X		3		X		X		3		X		X		3		X		X		3		X		X	
6					X	6					X	6					X	6					X	6					X
8						8						8						8						8					
9						9						9						9						9					

**Table 3:** The accuracy of the automated registration of scans for Test Site II (both scenarios): green, correct matching (full registration); orange, preliminary orientation requiring final registration using the ICP method; and red, no matching. “x” indicates no connection between scans (no overlap).

**Test Site II—Spherical Projection**																			
Scan no.	FAST			Scan no.	BRISK			Scan no	CenSurE			Scan no.	SURF			Scan no.	ASIFT		
	4	5	6		4	5	6		4	5	6		4	5	6		4	5	6
3				3				3				3				3			
4				4				4				4				4			
5				5				5				5				5			
**Test Site II—Orthoimages**																			
Scan no.	FAST			Scan no.	BRISK			Scan no.	CenSurE			Scan no.	SURF			Scan no.	ASIFT		
	4	5	6		4	5	6		4	5	6		4	5	6		4	5	6
3				3				3				3				3			
4				4				4				4				4			
5				5				5				5				5			
**Test Site II—Mercator Projection**																			
Scan no.	FAST			Scan no.	BRISK			Scan no.	CenSurE			Scan no.	SURF			Scan no.	ASIFT		
	4	5	6		4	5	6		4	5	6		4	5	6		4	5	6
3				3				3				3				3			
4				4				4				4				4			
5				5				5				5				5			

**Table 4:** The accuracy of the automated connecting of scans for Test Site III (both scenarios): green, correct matching (full registration); orange, preliminary orientation requiring the final registration using the ICP method; and red, no matching.

**Test Site III—Spherical Projection**																																							
Scan no.	FAST							Scan no.	BRISK							Scan no.	CenSurE							Scan no.	SURF							Scan no.	ASIFT						
	2	3	4	5	6	8	9		2	3	4	5	6	8	9		2	3	4	5	6	8	9		2	3	4	5	6	8	9		2	3	4	5	6	8	9
1								1								1								1								1							
2								2								2								2								2							
3								3								3								3								3							
4								4								4								4								4							
5								5								5								5								5							
6								6								6								6								6							
8								8								8								8								8							
**Test Site III—Orthoimages**																																							
Scan no.	FAST							Scan no.	BRISK							Scan no.	CenSurE							Scan no.	SURF							Scan no.	ASIFT						
	2	3	4	5	6	8	9		2	3	4	5	6	8	9		2	3	4	5	6	8	9		2	3	4	5	6	8	9		2	3	4	5	6	8	9
1								1								1								1								1							
2								2								2								2								2							
3								3								3								3								3							
4								4								4								4								4							
5								5								5								5								5							
6								6								6								6								6							
8								8								8								8								8							
**Test Site III—Mercator Projection**																																							
Scan no.	FAST							Scan no.	BRISK							Scan no.	CenSurE							Scan no.	SURF							Scan no.	ASIFT						
	2	3	4	5	6	8	9		2	3	4	5	6	8	9		2	3	4	5	6	8	9		2	3	4	5	6	8	9		2	3	4	5	6	8	9
1								1								1								1								1							
2								2								2								2								2							
3								3								3								3								3							
4								4								4								4								4							
5								5								5								5								5							
6								6								6								6								6							
8								8								8								8								8							

**Table 5:** The accuracy of the automated connecting of scans for Test Site IV (both scenarios): green, correct matching (full registration), orange, preliminary orientation requiring the final registration using the ICP method and red, no matching.

**Test Site IV—Spherical Projection**																																		
Scan no.	FAST						Scan no.	BRISK						Scan no.	CenSurE						Scan no.	SURF						Scan no.	ASIFT					
	7	8	9	10	11	12		7	8	9	10	11	12		7	8	9	10	11	12		7	8	9	10	11	12		7	8	9	10	11	12
6							6			X	X	X		6							6							6						
7							7							7							7							7						
8							8							8							8							8						
9							9							9							9							9						
10							10							10							10							10						
**Test Site IV—Orthoimages**																																		
Scan no.	FAST						Scan no.	BRISK						Scan no.	CenSurE						Scan no.	SURF						Scan no.	ASIFT					
	7	8	9	10	11	12		7	8	9	10	11	12		7	8	9	10	11	12		7	8	9	10	11	12		7	8	9	10	11	12
6							6							6							6							6						
7							7							7							7							7						
8							8							8							8							8						
9							9							9							9							9						
10							10							10							10							10						
**Test Site IV—Mercator Projection**																																		
Scan no.	FAST						Scan no.	BRISK						Scan no.	CenSurE						Scan no.	SURF						Scan no.	ASIFT					
	7	8	9	10	11	12		7	8	9	10	11	12		7	8	9	10	11	12		7	8	9	10	11	12		7	8	9	10	11	12
6							6							6							6							6						
7							7							7							7							7						
8							8							8							8							8						
9							9							9							9							9						
10							10							10							10							10						

**Table 6:** Comparison of results of the TLS image-based method and the target-based registration method.

**Detector**	**The Statistics of the Linear RMSE on Marked Check Points [mm]**			
	**Test Site I** **(Image-Based Approach)**			**Test Site I** **(Target-Based Approach)**
	**Cartographic Transformation**			
	**Spherical**	**Orthoimages**	**Mercator**	**Spherical**
FAST	4.2	6.7	5.8	5.7
BRISK	5.1	6.5	6.7	
CenSurE	4.6	6.6	5.6	
SURF	4.2	6.8	4.8	
ASIFT	4.8	6.2	5.2	
**Detector**	**The Statistics of the Linear RMSE on Marked Check Points [mm]**			
	**Test Site III** **(Image-Based Approach)**			**Test Site III** **(Target-Based Approach)**
	**Cartographic Transformation**			
	**Spherical**	**Orthoimages**	**Mercator**	**Spherical**
FAST	3.0	3.9	4.3	1.3
BRISK	4.4	5.6	4.1	
CenSurE	2.7	4.6	3.6	
SURF	4.1	5.9	4.7	
ASIFT	3.2	1.8	3.2	
**Detector**	**The Statistics of the Linear RMSE on Marked Check Points [mm]**			
	**Test Site IV** **(Image-Based Approach)**			**Test Site IV** **(Target-Based Approach)**
	**Cartographic Transformation**			
	**Spherical**	**Orthoimages**	**Mercator**	**Spherical**
FAST	29.3	13.3	-	3.8
BRISK	32.3	-	-	
CenSurE	32.9	-	25.2	
SURF	24.7	8.4	39.0	
ASIFT	10.1	29.3	11.2	

**Table 7:** Summary of evaluation of the usefulness criteria of point detection algorithms for all Test Sites according to the scale from 1 to 5 with the worst (red) and the best (green) results marked.

Evaluation Criteria	ASIFT	BRISK	CenSurE	FAST	SURF
Time of computation	1	4	5	3	2
The number of tie points	5	1	2	4	3
Percentage of correctly detected tie points	4	2	2	3	2
Deviation on control points—X axis	5	2	3	3	4
Deviation on control points—Y axis	5	2	3	4	3
Deviation on control points—Z axis	5	1	2	3	3
Deviation on natural check points—X axis	5	1	2	4	4
Deviation on natural check points—Y axis	5	2	2	3	3
Deviation on natural check points—Z axis	5	1	2	4	4
RMSE on marked check points—linear	5	1	2	3	4
Completeness	5	1	3	4	4
**Total**	50	18	28	38	36
**Final Ranking**	I	V	IV	II	III
